# Prehospital digital photography and automated image transmission in an emergency medical service – an ancillary retrospective analysis of a prospective controlled trial

**DOI:** 10.1186/1757-7241-21-3

**Published:** 2013-01-16

**Authors:** Sebastian Bergrath, Rolf Rossaint, Niklas Lenssen, Christina Fitzner, Max Skorning

**Affiliations:** 1 Department of Anaesthesiology, University Hospital Aachen, RWTH Aachen University, Pauwelsstr. 30, Aachen, D–52074, Germany; 2 Department of Medical Statistics, RWTH Aachen University, Aachen, Germany

**Keywords:** Telemedicine, Teleconsultation, Digital image, Emergency medical service, Picture transmission, Photo transmission

## Abstract

**Background:**

Still picture transmission was performed using a telemedicine system in an Emergency Medical Service (EMS) during a prospective, controlled trial. In this ancillary, retrospective study the quality and content of the transmitted pictures and the possible influences of this application on prehospital time requirements were investigated.

**Methods:**

A digital camera was used with a telemedicine system enabling encrypted audio and data transmission between an ambulance and a remotely located physician. By default, images were compressed (jpeg, 640 x 480 pixels). On occasion, this compression was deactivated (3648 x 2736 pixels). Two independent investigators assessed all transmitted pictures according to predefined criteria. In cases of different ratings, a third investigator had final decision competence. Patient characteristics and time intervals were extracted from the EMS protocol sheets and dispatch centre reports.

**Results:**

Overall 314 pictures (mean 2.77 ± 2.42 pictures/mission) were transmitted during 113 missions (group 1). Pictures were not taken for 151 missions (group 2). Regarding picture quality, the content of 240 (76.4%) pictures was clearly identifiable; 45 (14.3%) pictures were considered “limited quality” and 29 (9.2%) pictures were deemed “not useful” due to not/hardly identifiable content. For pictures with file compression (n = 84 missions) and without (n = 17 missions), the content was clearly identifiable in 74% and 97% of the pictures, respectively (p = 0.003). Medical reports (n = 98, 32.8%), medication lists (n = 49, 16.4%) and 12-lead ECGs (n = 28, 9.4%) were most frequently photographed. The patient characteristics of group 1 vs. 2 were as follows: median age – 72.5 vs. 56.5 years, p = 0.001; frequency of acute coronary syndrome – 24/113 vs. 15/151, p = 0.014. The NACA scores and gender distribution were comparable. Median on-scene times were longer with picture transmission (26 vs. 22 min, p = 0.011), but ambulance arrival to hospital arrival intervals did not differ significantly (35 vs. 33 min, p = 0.054).

**Conclusions:**

Picture transmission was used frequently and resulted in an acceptable picture quality, even with compressed files. In most cases, previously existing “paper data” was transmitted electronically. This application may offer an alternative to other modes of ECG transmission. Due to different patient characteristics no conclusions for a prolonged on-scene time can be drawn. Mobile picture transmission holds important opportunities for clinical handover procedures and teleconsultation.

## Background

Digital cameras are becoming increasingly common in emergency departments and emergency medical services (EMS). Potential applications include photo documentation, facilitation of handover procedures, and collecting clinical pictures for teaching purposes. Medical societies, like the British Orthopaedic Association and the British Association of Plastic Surgeons, recommend the photo documentation of open wounds [1]. In Germany, digital cameras are mandatory on physician-staffed advanced life support ambulances and response units [2]. However, few studies to date have assessed the use of digital cameras in emergency medicine. Morgan et al. reported in 2007 that 80% of the analysed emergency departments in the United Kingdom had a digital or Polaroid camera that was ready for operation [3]. The availability of photographic equipment has increased over the past decade, but little data have been published about the scenarios in which digital photography was used, content of photographs taken and reasons for the use of digital photography in emergency medicine [4,5].

The objective of this study was to investigate the feasibility of using a digital camera for still picture transmission in a prehospital telemedicine system on one specifically equipped ambulance. The picture quality was rated, and the content of the pictures was classified. Moreover, the study analysed how often and for which diagnoses the camera was used. The possible influence of this application on prehospital time requirements was also evaluated.

## Methods

The study was conducted as a retrospective, additional data analysis of data that was gathered during a previously performed prospective, controlled trial in the context of the research project entitled “Med-on-@ix” in Aachen, Germany [6-8].

### Telemedicine system and approach within the prospective controlled trial

From May to September in 2010 289 emergency missions were performed in total by one advanced life support ambulance on weekdays between 7.30 am and 4 pm. In addition to the two paramedics who were normally assigned to this unit, the vehicle was staffed by an additional EMS physician during this funded period. Moreover, the vehicle was equipped with a portable telemedicine system that allowed real-time vital data transmission, 12-lead electrocardiogram (ECG) transmission on demand and video transmission (i.e. from a video camera embedded into the ceiling of the ambulance). During the development phase of the system we found out that mobile video transmission from a video camera that was fixed on a headband was not meaningful, because the perspective changed permanently due to head movements of the EMS personnel. Furthermore, video transmission required a stable broadband data connection, which was easier to realise with the roof antennas of the ambulance. Therefore, a mobile digital camera (Powershot A1000IS, Canon Inc, Tokyo, Japan) was used to take and transmit still pictures. A portable data transmission unit was developed (peeq-Box, P3 communications, Aachen, Germany) that enabled an encoded broadband communication via four parallel data channels from different network providers (each data channel enabled the use of second or third generation mobile networks; max. uplink 1.4 Mbit/s, max. downlink 6 Mbit/s) and voice communication between the EMS team and a teleconsultation centre. This centre was staffed with experienced EMS physicians (tele-EMS physicians), who offered medical and organisational support, when required. If a picture was taken by the EMS team, it was automatically transmitted from the digital camera to the transmission unit via a wireless LAN (Eye-Fi card, Eye-Fi, Mountain View, CA, USA) and then transferred to the teleconsultation centre via mobile networks. By default, the system compressed the photos to a file size of < 100 kB (jpeg format, 640 × 480 pixels). This compression mode was deactivated on some study days in order to gain experience with the transmission of uncompressed picture files (3648 × 2736 pixels, approximate file size 2–2.5 MB). Under ideal testing circumstances, end-to-end transmission times were approximately 25 seconds when using the compression mode and up to 2 minutes with uncompressed files. EMS teams were free to decide which telemedical applications they wanted to use based on medical necessity. In fact, no defined criteria were established to dictate when picture transmission should or should not be used, but in a previously performed training program all EMS physicians and paramedics were cautioned to be judicious when gathering data. More specifically, they were told that pictures should only be taken and transmitted when a medical rationale supported the action. During the training examples of situations when digital image transmission might be meaningful were distributed: e.g., transmission of medical reports, medication lists, medication packages, accident kinematics, open wounds or skin rashes and facial asymmetry. Particular emphasis was given to the high sensitivity related to pictures that allow the patient to be identified (e.g., patient’s face, name and date of birth on documents). No technical guidelines for the use of the digital camera were provided to this group. A questionnaire about the use and performance of the telemedical applications was completed after each mission.

### Ethics statement and trial registration

This study exhibits an ancillary, retrospective data analysis of transmitted pictures and EMS mission data that were gathered during a prospective, controlled trial that was registered (http://www.controlled-trials.com/isrctn/pf/83270177). It was approved by the local ethics committee (University Hospital Aachen, Germany, registration number EK 141/09). The scientific analysis of transmitted pictures was specifically approved in the statement. In two previously obtained legal opinions the operation of the system was in accordance with national law. All patients were informed about the telemedical data transmission, and informed consent was obtained. All data recorded within the project were strictly for non-commercial means, and the recorded digital images were only stored for documentation and scientific purposes.

During the prospective, controlled trial we found that in acute stroke the telemedical approach led to improved data transmission from the prehospital to the in-hospital setting. Prehospital time intervals were not affected negatively by teleconsultation and the quality of prehospital stroke diagnoses was comparable between telemedically supported EMS and regular EMS [7]. In acute coronary syndromes significantly more patients received urgent percutaneous coronary intervention in the telemedicine group [8].

### Retrospective ancillary data collection and analysis

**Figure 1:**
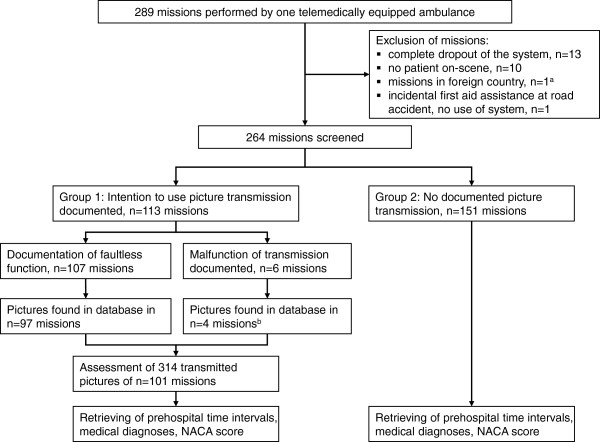
**Trial Flow.** NACA score, National Advisory Committee for Aeronautics Score (seven-ary severity score). ^a^: Mission in the Netherlands, EMS station was 7.3 km away from the border. Cross-border assistance between the EMS is governed by contract. ^b^: In 4 missions mission related pictures were found, although a malfunction of this application was documented.

Prior to the analysis, a local expert committee consisting of EMS physicians defined criteria for the evaluation and classification of the pictures (Tables 1 and 2). All questionnaires were screened to detect missions where digital photography and transmission was documented (Figure 1). The identified missions were searched in a specifically secured database, where all data of the missions were stored. Two faculty investigators had a secured access to the database for a limited time. Both assessed the missions’ data and all pictures independently.

**Table 1:** Classification of transmitted still pictures

**Content**	**Number of transmitted pictures (% of categorized photos)**	**Number of corresponding emergency missions**
Medication list	49 (16.4%)	37
Medication packages	12 (4%)	6
Medical report, physician’s note	98 (32.8%)	42
Nursing report	17 (5.7%)	11
12-lead ECG	28 (9.4%)	13
Patient (with identifiable face)	16 (5.4%)	12
Patient’s detail (without identifiable face)	20 (6.7%)	10
Surrounding of emergency site	10 (3.3%)	5
Accident kinematics/mechanism	2 (0.7%)	1
Other content (not previously defined)	47 (15.7%)	30

**Table 2:** Diagnosis related usage of picture transmission

**Diagnosis**	**Number of missions with intention to use picture transmission (n = 113) [*Mean number of transmitted pictures ± SD*]**	**Number of missions without picture transmission (n = 151)**	**p-value**
Acute coronary syndrome	24 [*3.33 ± 3.38*]	15	0.014
Cardiopulmonary resuscitation successful	2 [*7.0 ± 9.90*]	1	-^d^
Cardiopulmonary resuscitation not successful	0 [−]	1	-^d^
Other cardio-circulatory emergency	21 [*2.48 ± 1.47*]	23	0.51
Stroke^a^/TIA	11 [*3.82 ± 2.14*]	6	0.076
Seizure and other neurological emergency	13 [*1.85 ± 0.99*]	15	0.69
Syncope/orthostatic dysregulation	11 [*2.73 ± 2.05*]	18	0.69
Minor and moderate trauma	8 [*2.25 ± 1.83*]	11	1.0
Major trauma	0 [−]	1	-^d^
Determination of death^b^	1 [−]	8	0.083
Paediatric emergency^c^	2 [*2.50 ± 2.12*]	5	0.70
Asthma/obstructive airway disease	2 [*1.50 ± 0.71*]	6	0.47
Psychiatric emergency	2 [*3.00 ± 1.41*]	3	1.0
Intoxication	1 [−]	4	0.40
Other emergency	15 [*2.27 ± 1.62*]	34	0.078

*SD*, standard deviation; *TIA*, transient ischaemic attack.

^a^intracranial haemorrhage included.

^b^no resuscitation attempts.

^c^defined as age < 6 years.

^d^statistical comparison not meaningful due to small case numbers.

The quality of the pictures were assessed by the two faculty investigators and categorised into one of three classes. If the content of a picture was clearly identifiable (e.g., text clearly readable) the photograph was rated as “good quality.” The rating “limited quality” was assigned to pictures with a limited clarity of the content. Pictures were evaluated as “not useful,” if the content was not or only partially identifiable. Additionally, the resolution and file size of each picture was retrieved for analysis to determine if the file size influenced the recognisability or readability of the pictures.

The content of each picture was categorised (Table 1). If a picture of a medical record included a medication list, it was assigned to the category “medical record” because nearly all medical records contained recommendations for medications. After the first assessment, all datasets that were rated differently by the investigators were evaluated by a third investigator, who discussed the situation with investigators 1 and 2 until either a joint decision was made or a final decision was made by investigator 3. Medical diagnoses and demographic data were obtained from the EMS protocol sheets for all missions with (group 1) and without documented picture transmission (group 2). All missions within both groups were performed by the same ambulance and medical personnel (Figure 1). The time intervals designated as “on-scene time” and “ambulance arrival to hospital arrival” were measured with electronic timestamps that were transmitted via radio from the ambulance to the EMS dispatch centre; a comparison of the time data from each group was completed. All data were transferred to a database (SAS database, Version 9.2, SAS Institute Inc., Cary, NC, USA).

### Statistical methods

Categorical data are presented as frequencies and percentages. The numbers of transmitted pictures and file sizes are expressed as means and standard deviations (±SD), whereas age, NACA scores and time intervals are expressed as medians and interquartile ranges (IQR). To analyse the influence of the file size on the picture quality for missions with activated compression mode and missions without file compression, ratios of “pictures with good quality/total number of pictures” were calculated and compared using the unpaired Wilcoxon test. Ages, NACA scores and prehospital time intervals of group 1 were compared to those of group 2 using the unpaired Wilcoxon test. Gender distribution and the frequency of medical diagnoses were compared using Fisher’s exact test. Due to the fact that this was a pilot study, no alpha-adjustment was performed and p-values of less than 0.05 were considered to be significant. All statistical analyses were conducted using SAS (Version 9.2, SAS Institute Inc, Cary, NC, USA).

## Results

### Usage of digital image transmission and picture quality

**Figure 2:**
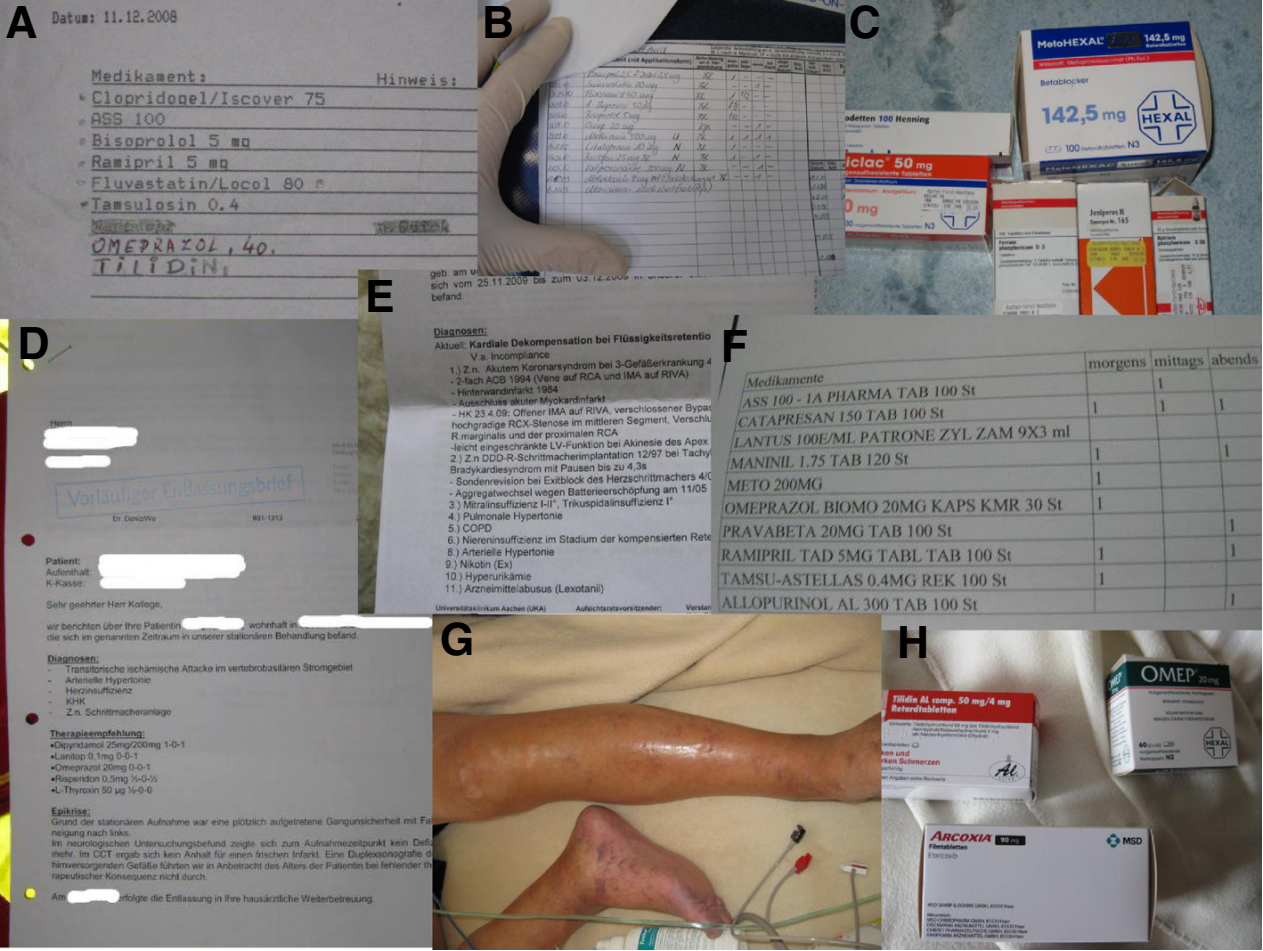
**Sample pictures.** Only pictures without identifiable information are displayed. Patient names and patient related details were obscured. **A**: Medication list, **B**: Medication list, **C**: Medication packages, **D**: Medical report, **E**: Medical report, **F**: Medication list, **G**: Patient’s detail, **H**: Medication packages.

**Figure 3:**
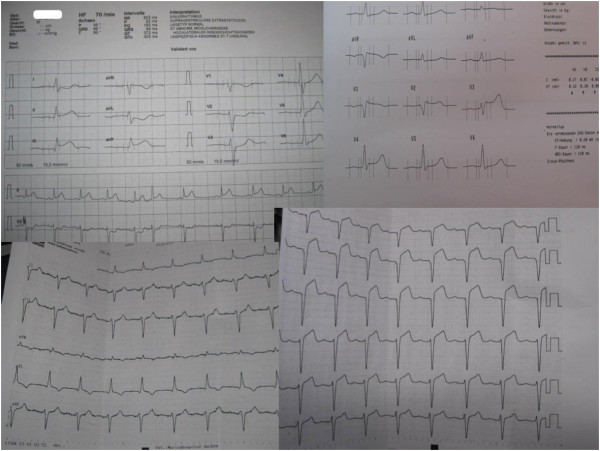
**Sample 12-lead ECGs.** Only ECGs without identifiable personal data are displayed.

Overall 264 missions were screened, and 314 pictures for 113 (42.8%) missions (range 0–17, mean 2.77 ± 2.42 pictures in each mission) were found in the database and were included into the analysis (Figure 1). Of them 240 (76.4%) pictures (mean 2.38 ± 1.97 pictures per mission) exhibited a clearly identifiable content (“good quality”). A total of 45 (14.3%) pictures (mean 0.45 ± 1.08 pictures per mission) were of “limited quality” and 29 (9.2%) pictures were assessed as “not useful”, because their content was not or only hardly identifiable. Common reasons for an unacceptable picture quality included unintentional camera shaking or the use of the flash in situation with a bright background, especially with photos of paper-based documents. Figures 2 and 3 show examples of transmitted pictures without identifiable personal data.

### Comparison of compressed and uncompressed file transmission

In 84 of the missions with successful transmission, file compression was activated, and in 17 missions photographs were transmitted without compression. The mean file size was 45 ± 34 kB and 2261 ± 300 kB with the lower and higher resolutions, respectively. With the compression mode activated, 74% of the pictures contained clearly identifiable content according to the faculty investigators’ assessment (median ratio “pictures with good quality/total number of pictures” =100%, range 50%-100%, n = 84) as compared to 97% without file compression (median ratio “pictures with good quality/total number of pictures” =100%, range 0%-100%, n = 17), p = 0.003.

### Classification of pictures and emergency missions

Overall, 299 pictures were classified according to the predefined content criteria (Table 1). All other pictures (n = 15) were not classifiable due to unidentifiable content. Most frequently, medical reports and physician’s notes were photographed (n = 98, 32.8%). The content of 47 pictures did not fit one of the nine predefined categories. Most of these pictures were photographed personal data (e.g., patient’s name and address). Table 2 displays the diagnoses related to the digital image transmission. In situations with acute coronary syndromes, other circulatory emergencies, cardiopulmonary resuscitation, and neurological emergencies, picture transmission was used in 46 to 67% of the corresponding missions.

Patient characteristics of group 1 (i.e., those missions with documented picture transmission, n = 113) were compared to those of group 2 (i.e., missions without picture transmission, n = 151). The median patient age in group 1 was 72.5 (IQR 30) years as compared to 56.5 (IQR 41) years in group 2, p = 0.001. No significant difference in terms of gender distribution was detected; more specifically, 47.8% (n = 54) versus 49.3% (n = 71) were female, p = 0.90. The median NACA scores were 4 (IQR 1) for group 1 versus 3 (IQR 1), p = 0.28. No significant differences regarding the frequency of the diagnoses were found between the groups except for acute coronary syndromes (Table 2).

### Prehospital time requirements

The median on-scene time was 23 min (IQR 10, n = 205 with complete documentation of time interval), and the median ambulance arrival to hospital arrival time was 35 min (IQR 14, n = 198 with complete documentation of time interval) for all missions. For group 1 (n = 93 with complete documentation), the median on-scene time was 26 min (IQR 9) as compared to 22 min (IQR 11) for group 2 (n = 112 with complete documentation), p = 0.011. The median ambulance arrival to hospital arrival times for groups 1 and 2 were 35 min (IQR 11, n = 90 with complete documentation of time interval) and 33 min (IQR 14.5, n = 108 with complete documentation), respectively (p = 0.054).

## Discussion

This study evaluated the quality and content of transmitted digital images in a prehospital telemedicine system under routine clinical conditions. Pictures were taken and transmitted in 42.8% of the analysed missions. Most of them were deemed of “good quality” (76.4%) and mainly captured previously existing documents, like medical reports, medication lists, or 12-lead ECGs. In only 6.1% of the pictures, an identifiable face of the patient was displayed, but most of the documents contained confidential personal data. Between the groups, patient characteristics differed significantly in terms of age and the frequency of acute coronary syndromes. Therefore, no conclusions from a comparison of these groups should be drawn regarding the influence of image transmission on prehospital time intervals.

Digital image transmission was used frequently and resulted in the transmission of photographs with predominantly satisfactory picture quality. Camera shaking, which was a common reason for impaired quality, is nearly impossible to prevent in an emergency setting. Using the flash while taking pictures of documents frequently caused pictures to be overexposed, thereby resulting in pictures without an identifiable content. Therefore, we recommend adjusting the default settings of the flash used. Quality and readability improved significantly when the uncompressed transmission mode was used, but the file sizes were about 50 times higher. Compressed jpeg-files seem to be reasonable in this setting, but studies to evaluate whether lesser file compression improves the quality in the same way as the uncompressed mode are needed. Especially in rural areas, the amount of data is crucial in terms of determining acceptable transmission times because mobile networks with lower uplink capacities are mostly available.

Most of the photos contained personal data, but an identifiable face was photographed in only a few missions. In most instances, no new data was generated (e.g., picture of a face); instead, previously existing data (e.g., medical documents) were converted into a digital format and transmitted. To transmit video files from the emergency site could have been an alternative, but technical and practical arguments like described above led to the use of still picture transmission. In the future, when mobile networks enable faster and more stable upload capacities, mobile video transmission might be easier to realise and could be more meaningful in certain situations (e.g., remote neurological assessment). However, the focus of the described telemedicine system was to achieve sufficient data for remote teleconsultation within a narrow time period. Most of the time documents were photographed and such information can be extracted easily from single pictures. Thus, the tele-EMS physician was able to gain detailed information without committing to an overly time consuming audio communication with the EMS team. To get sufficient data for necessary medical decisions within the shortest possible time period is crucial for emergency teleconsultation. Unfortunately, the extent to which measurable benefits to patient care were reached remains unclear. If electronic patient records become accessible via the Internet in the future or if localized electronic health information (e.g., electronic health card) becomes routinely used, the need for photographs of medical documents will be reduced noticeably. The described system enabled image transmission of previously printed 12-lead ECGs (e.g., by the general practitioner) in addition to or instead of the 12-lead ECG transmission from the vital data monitor. Consequently, this data transmission enabled the pre-notification of the cardiologist on-call when needed. Patients with acute coronary syndromes that were treated with additional teleconsultation received significantly more often urgent percutaneous coronary intervention compared to patients that were treated by regular EMS [8]. Previous research has clearly demonstrated that transmission of the 12-lead ECG enhances treatment processes and improves outcome [9-13]. In a study using a cellular video-phone for remote interpretation of prehospital 12-lead ECGs, the interpretation quality was comparable to the evaluation of a printed ECG [14]. Ohtsuka et al. demonstrated that the transmission of 12-lead ECGs was feasible and rapid using an older type camera phone [15]. All pictures of ECGs (n = 28) in our study were completely readable (Figure 3). In situations where a previously recorded ECG differs from the current ECG, picture transmission seems to offer a meaningful contribution to the standard ECG transmission modes. However, the main intention of picture transmission was to enable rapid medical assistance by a remote experienced physician who was the receiver of all transmitted data. Medical decisions can be based on transmitted pictures if they contain critical information (e.g., medical report, 12-lead-ECG) and this approach can reduce the amount of audio communication needed during emergency teleconsultation.

To evaluate the influence of picture transmission on prehospital time requirements, we first analysed the comparability of both groups regarding patient characteristics. Although no significant differences in the NACA score and gender distribution were detected, patients of group 1 were significantly older and significantly more patients were diagnosed with acute coronary syndrome compared to group 2. Therefore, no conclusions from a significantly prolonged on-scene time should be drawn due to the different patient characteristics that may have caused the prolongation of the on-scene time. Overall, the meaningfulness of time interval comparisons between both groups is questionable. However, the ambulance arrival to hospital arrival intervals did not differ significantly, but data about the exact driving route or the use of emergency lights and sirens on route to the hospital were not available.

The operation of the described system represents a considerable effort with associated costs. In contrast, the use of smartphones could be a comparatively inexpensive alternative. Indeed, pictures taken with a smartphone can be transmitted to any e-mail address or to another smartphone. Unfortunately, this transmission occurs mostly without proper encryption, and the reliability is unknown. Furthermore, local storage of confidential data seems to be problematic. Smartphones have already been evaluated for similar purposes in different disciplines. For example, in the plastic surgery context, image transmission led to shorter treatment intervals with a comparable diagnostic accuracy in the assessment of free flaps when compared to the classic in-house assessment [16]. Even with an older 1.1 megapixel camera phone, satisfactory assessments of the replantation potential of completely amputated fingers were possible [17]. Pirris et al. demonstrated that a patient’s cell phone camera can be used for the remote evaluation of infected wounds [18]. In such an ambulatory setting, ultra-short transmission times are not required, and in situations with non-urgent communication between a patient and the surgeon, compromises in the reliability of transmission are acceptable. However, for prehospital teleconsultation a single smartphone does not enable multiple telemedical applications. Indeed, secure availability, high reliability, and encoded transmission are required for teleconsultation in EMS. Our developed pilot system was designed to meet these demands, but in 4.5% of all missions (n = 289), a complete drop-out occurred. Prior to a routine use the reliability must be improved in order to achieve the advantage offered by the combined use of several networks. Successful functioning is crucial for the introduction of potentially helpful telemedical applications. Prior to our project, different systems with similar applications were developed but not evaluated in clinical routine [19]. In a previous observational study with a precursor of our system, a tablet-computer with a built-in camera was used. The frequency of picture transmission was comparable; however the content of the photos was not evaluated, and the picture quality was only rated cumulatively [20]. Poor reliability and stability of this tablet-PC led to the described changes.

In addition to the operation of digital cameras within a telemedicine system, they can be useful for documentation and teaching in EMS. Photos taken on-scene enable an in-hospital trauma team to get realistic impressions of accident kinematics. A large display of images is desirable, but even the built-in screen of the camera allows this information to be distributed. Prehospital images should be saved in picture archiving and communication systems (PACS) so that they become available for the whole treatment process.

### Limitations

The frequency of picture transmission probably depended not only on the medical necessity but also on the team’s attitude towards the system. Pictures were taken during day-time from spring to autumn due to restricted funding capacities. Pictures taken during the night or under different weather conditions (e.g., snow) would probably result in varying picture quality. Unfortunately, no technical guidelines for camera use were provided to the EMS. If such recommendations would have been implemented, different results may have been detected. As mentioned above, the non comparable groups led to very limited assessable results regarding the comparison of time intervals. Moreover, incorrect assessments by the investigators cannot be ruled out definitely, but we minimised this risk by using the described assessment procedure. This study was designed to evaluate feasibility, quality, and content of transmitted images and influences on time requirements. Influences on the patient outcomes were not measured and not purpose of this study. If the described approach would be implemented into routine care to support paramedic staffed ambulances this study has to be repeated in this different setting. The indications for still picture transmission might be different when no physician in on-scene.

## Conclusions

Encrypted picture transmission in EMS missions is feasible. A remotely located physician received photos with high informative value. Picture quality was satisfactory, but the optimal file compression mode remains unclear and should be adjusted according to local network availability. In most cases previously existing medical documents or 12-lead ECGs were photographed. Image transmission of printed 12-lead ECGs seems to be an alternative to standard transmission modes for special situations. Nevertheless, the influence of picture transmission on prehospital time requirements remains unclear. Although digital photography in medicine is cheap and easy to realize, secured data storage must be guaranteed. Further studies to evaluate the influence of this application on patient outcome should be conducted.

## Competing interests

The study was conducted within the joint research project “Med-on-@ix” funded by the German Federal Ministry of Economics and Technology (BMWi), Project-No.: 01 MB07022. Philips Healthcare (Hamburg, Germany) and P3 communications (Aachen, Germany) brought in their own financial resources. The funders had no role in the study design, data collection and analysis, decision to publish, or preparation of the manuscript. No author had financial relationships or other non-financial dependencies with the funding sponsors.

## Author’s information

Sebastian Bergrath: http://www.anaesthesie.ukaachen.de; http://www.medonaix.de

## Authors’ contributions

SB, MS and RR had the initial idea of the study. SB, NL and MS assessed and rated the pictures. CF and SB performed the statistical analysis. SB, RR, NL, CF and MS performed data interpretation. SB, RR, NL and MS drafted the manuscript and SB, NL and MS performed literature search. MS, CF, RR and NL revised the manuscript critically. All authors read and approved the final manuscript.
